# Longitudinal health-related quality of life assessment: implications for prognosis in ovarian cancer

**DOI:** 10.1186/1757-2215-6-17

**Published:** 2013-03-08

**Authors:** Digant Gupta, Donald P Braun, Edgar D Staren, Maurie Markman

**Affiliations:** 1 Cancer Treatment Centers of America®, 1336 Basswood Road, Schaumburg, IL, 60173, USA

## Abstract

**Background:**

There is no information in the literature on the impact of changes in quality of life (QoL) scores on prognosis in ovarian cancer. We investigated whether changes in QoL during treatment could predict survival in ovarian cancer patients.

**Methods:**

We evaluated 137 ovarian cancer patients treated at our institution between Jan 2001 and Dec 2009 who were available for a minimum follow-up of 3 months. QoL was evaluated at baseline and after 3 months of treatment using EORTC-QLQ-C30. Cox regression evaluated the prognostic significance of baseline and changes in QoL scores after adjusting for clinical and demographic variables.

**Results:**

Associations between changes in QoL and survival were observed for global function, appetite loss and constipation. Every 10-point increase (improvement) in global function from baseline to 3 months was associated with a 10% decreased risk of death (HR=0.90; 95% CI: 0.81 to 0.99, p=0.03). The corresponding HRs for 10-point increase (deterioration) in appetite loss and constipation scales were 1.20 (95% CI: 1.06 to 1.35; p=0.005) and 1.13 (95% CI: 1.02 to 1.24; p=0.02) respectively.

**Conclusions:**

This exploratory study provides evidence that an improvement in appetite, constipation and global health scores during the first 3 months of treatment is significantly associated with improved survival time in ovarian cancer. These findings justify serial, systematic assessment of global health, appetite and constipation in ovarian cancer patients being treated, and suggest that modalities designed to improve these functions may be beneficial clinically.

## Background

With an incidence of about 26,000 cases per year, ovarian cancer is the second most common gynecologic malignancy and the leading cause of mortality from gynecologic cancers in the USA, resulting in approximately 14,500 deaths annually [1]. Most women are diagnosed as having advanced stage disease and undergo aggressive debulking surgery and primary postoperative chemotherapy, and yet the majority experience recurrence [2-4]. Tumor stage, age, performance status, tumor histology, and residual tumor are some of the independent predictors of survival in patients with ovarian cancer [5].

Given that ovarian cancer and its treatment can cause significant physical and psychological morbidity [3], assessment of quality of life (QoL) is particularly important for these patients [6-10]. It is important to focus not only on the short-term side effects of treatment, but also on the effects of treatment on symptoms and functional status during periods of disease remission, relapse, and survival. Such effects not only influence a patient’s overall QoL, they also influence the ability to tolerate additional salvage therapy for extended periods of time with the potential of delaying disease progression [11].

QoL is a multidimensional construct that includes physical, social, psychological and functional domains at the very least. A growing consensus among health care providers and researchers is that treatment efficacy should be judged by effects on both quantity and quality of life. This has led to the inclusion of QoL assessment as a primary endpoint in cancer clinical trials along with traditional endpoints of tumor response and survival. QoL measurements provide information about the impact of the disease and its treatment on multiple patient parameters and can aid physicians in selecting and managing antineoplastic and supportive therapy [10].

Baseline or pretreatment QoL has been shown to be a prognostic indicator in ovarian cancer in three previously published studies [12-14]. However, to the best of our knowledge, there is no study in the literature investigating the prognostic impact of changes in QoL scores on survival in ovarian cancer, whether this is assessed at the time of initial cancer diagnosis or following disease relapse. In the current study, we investigated whether pretreatment QoL parameters as well as changes in these same QoL scores from baseline to 3 months post-initiation of therapy could predict survival in patients with ovarian cancer. This study builds upon our previous work in this area investigating the relationship between changes in QoL and survival in other types of cancers [15,16].

## Methods

### Study population

We examined 137 histologically confirmed ovarian cancer patients treated at Cancer Treatment Centers of America® at Midwestern (MRMC) and Southwestern (SRMC) Regional Medical Centers between Jan 2001 and Dec 2009 who had QoL data available at both baseline and 3-month follow-up. The inclusion criteria for participation in this study were a histological diagnosis of ovarian cancer and the ability to read English. Patients with all stages of ovarian cancer were eligible for the study. Patients were excluded if they were unable to give informed consent or were unable to understand or cooperate with study conditions.

A trained clinical coordinator was responsible for determining eligibility, describing the study, and obtaining informed consent. All patients were assured that refusal to participate would not affect their future care in any way. Patients who chose to participate were presented with the questionnaire at their initial visit and instructed to return their completed questionnaires to the clinical coordinator within 24 hours. Thus, patients completed baseline questionnaires prior to receiving therapy at our facility. Following the completion of the baseline questionnaire, all patients were treated with an integrative model combining surgery, radiation and chemotherapy as appropriate, plus complementary therapy consisting primarily of nutritional, psychosocial, and spiritual support, naturopathic supplements, pain management, and physical therapy/rehabilitation. Patients completed the questionnaire again after a follow-up period of 3 months.

Additional patient data recorded for this study was age at presentation (current age), stage of disease at diagnosis and prior treatment history (previously treated versus newly diagnosed). The only follow-up information required was the date of death or the date of last contact/last known to be alive, obtained from the tumor registries at Midwestern and Southwestern Regional Medical Centers. This study was approved by the Institutional Review Board at Cancer Treatment Centers of America®.

### QoL assessment

QoL was assessed using the European Organization for the Research and Treatment of Cancer Quality of Life Questionnaire (QLQ-C30), which emphasizes a patient’s capacity to fulfill the activities of daily living. The QLQ-C30 is a 30-item cancer specific questionnaire that incorporates five functioning scales (physical, role, cognition, emotional, and social), eight symptom scales (fatigue, pain, and nausea/vomiting, dyspnea, insomnia, loss of appetite, constipation, diarrhea), financial well-being scale and a global scale (based on two items: “how would you rate your overall health during the past week” and “how would you rate your overall quality of life during the past week”). The raw scores are linearly transformed to give standard scores in the range of 0–100 for each of the functioning and symptom scales. Higher scores in the global and functioning scales and lower scores in the symptom scales indicate better QoL. A difference of 5–10 points in the scores represents a small change, 10–20 points a moderate change and greater than 20 points a large, clinically significant change from the patient’s perspective [17]. This instrument has been extensively tested for reliability and validity [18-20].

### Statistical analysis

Patient survival was the primary end point and defined as the time interval between the date of first patient visit to the hospital and the date of death from any cause or the date of last contact/last known to be alive. Two separate analyses were performed. First, the relationship between baseline QoL and patient survival was investigated for 137 patients. Second, the relationship between change in QoL scores between baseline and 3 months and survival was assessed for the same patient cohort. Change scores were calculated by subtracting the baseline scores from the 3-month QoL scores. The overall survival was calculated using the Kaplan-Meier method. Clinical and QoL variables were evaluated using univariate Cox proportional hazards models to determine which parameters showed individual prognostic value for survival. Multivariate Cox proportional hazards models were then performed to evaluate the joint prognostic significance of all QoL and clinical factors. Each QLQ-C30 scale was treated as a continuous variable for the purpose of Cox regression analyses. The effect of QoL parameters on patient survival was expressed as hazard ratios (HRs) with 95% confidence intervals (CIs). Changes of 10 or more points on a 0 to100 scale are considered clinically relevant [21], so we present hazard ratios for a 10-point change on the continuous QoL variables. An effect was considered to be statistically significant if the p value was less than or equal to 0.05. All statistical tests were two sided. All data were analyzed using IBM SPSS version 20.0 (Chicago, IL, USA).

Cox regression with time-invariant covariates assumes that the ratio of hazards for any two groups remains constant in proportion over time. We checked this assumption by first examining log-minus-log plots for the categorical predictors and then fitting a Cox regression with a time-varying covariate for each predictor in turn. Potential multicollinearity was assessed in two steps. Large values (above 0.75) of Pearson’s correlation coefficients were used as an initial screen for pairs of QoL variables. As a second check, the variance inflation factor was used with the final model to verify that multicollinearity was not significantly influencing model coefficients [22,23]. In order to minimize instability of the final multivariate model resulting from high multicollinearity, global QoL was evaluated separately because it is most highly correlated with all other variables on the QLQ-C30 questionnaire, and also because it is difficult to interpret and manipulate clinically. Finally, to assess the possible influence of sample bias on the results, as well as to investigate the stability of the model coefficients, we performed a bias-corrected and accelerated (BCa) bootstrap resampling procedure. We generated 1000 samples, each the same size as the original data set, by random selection with replacement. Cox regression was then run separately on these 1000 samples to obtain robust estimates of the standard errors of coefficients, and hence the p values and 95% BCa CIs of the model coefficients [24].

## Results

### Patient characteristics

Table 1 describes the baseline characteristics of our patient cohort. All newly diagnosed patients received appropriate de-bulking surgery followed by a platinum-based chemotherapy. All patients with recurrent disease received systemic chemotherapy chosen by their attending physician based on prior treatment history. Table 2 displays the baseline QoL scores across the two categories of prior treatment history. There were no statistically significant differences in QoL scores between newly diagnosed and previously treated patients.

**Table 1:** Baseline characteristics of 137 ovarian cancer patients

**Characteristic**	**Categories**	**Number**	**Percent**
Age at diagnosis (years)	▪ Mean	51.1	
	▪ Median	51.0	
	▪ Range	28-75	
Vital status	▪ Dead	49	35.8
	▪ Alive	88	64.2
Treatment history	▪ Newly diagnosed	28	20.4
	▪ Previously treated	109	79.6
Stage at diagnosis	▪ Stage I	16	11.7
	▪ Stage II	15	10.9
	▪ Stage III	78	56.9
	▪Stage IV	28	20.4

**Table 2:** Baseline quality of life scores stratified by prior treatment history

**Baseline variable**	**Newly diagnosed**	**Previously treated**	**P**
	**(N = 28)**	**(N = 109)**	
	**Mean (standard deviation)**	**Mean (standard deviation)**	
**General quality of life**			
Global	58.9 (29.7)	60.0 (22.7)	0.83
**General function**			
Physical	76.1 (22.2)	76.8 (23.0)	0.89
Role	61.9 (32.9)	68.1 (31.1)	0.34
Emotional	68.4 (26.8)	70.6 (23.0)	0.66
Cognitive	79.1 (25.9)	78.1 (22.1)	0.83
Social	63.6 (33.9)	67.2 (32.7)	0.60
**General symptom**			
Fatigue	42.8 (30.7)	39.3 (27.8)	0.56
Nausea/vomiting	11.3 (23.5)	13.6 (22.2)	0.63
Pain	25.0 (29.2)	29.3 (28.4)	0.47
Dyspnea	17.8 (26.4)	22.3 (28.3)	0.45
Insomnia	33.3 (31.4)	39.7 (34.6)	0.37
Appetite loss	28.5 (41.2)	18.6 (25.0)	0.10
Constipation	15.4 (26.4)	23.8 (29.4)	0.17
Diarrhea	11.9 (24.3)	15.9 (24.6)	0.44
Financial	29.7 (34.3)	34.5 (32.3)	0.49

Table 3 describes the results of univariate Cox regression analysis for baseline patient characteristics. Treatment history was significantly associated with survival while age and stage at diagnosis were not. Median overall survival for the entire patient cohort was 33.5 months (95% CI: 11.5-55.6 months). The median survival for newly diagnosed and previously treated disease was 66.4 and 19.6 months respectively, log-rank p<0.001, which is not surprising given that previously treated patients had advanced stage/recurrent disease at the time of presentation to our institution.

**Table 3:** Baseline characteristics and associated HRs for death

**Characteristic**	**HR (95% CI)**	**P**
Age at diagnosis (years) used as continuous variable*	1.05 (0.78 – 1.33)	0.70
Stage at diagnosis (stages I-II as reference)	1.68 (0.83 – 3.39)	0.15
Treatment history (newly diagnosed as reference)	4.4 (1.9 – 10.0)	<0.001

*HRs correspond to a 10-point increment for age.

### Association between baseline quality of life and survival

Table 4 describes the baseline scores for all dimensions of EORTC QLQ-C30 instrument. Among the EORTC QLQ-C30 functioning scales, social functioning had the lowest (worst) mean score of 66.5 while the highest (best) mean score of 78.3 was recorded for cognitive functioning. Among the EORTC QLQ-C30 symptom scales, nausea/vomiting had the lowest (best) mean score of 13.1 while the highest (worst) mean score of 40.0 was recorded for fatigue. Table 4 also displays the results of univariate and multivariate Cox regression analyses for each QoL variable. The HRs along with their 95% CIs for every 10-point increase in all EORTC QLQ-C30 scales are given. On univariate analysis, no baseline QoL variables were predictive of survival. Before proceeding with multivariate analysis, we checked the bivariate Pearson’s correlation among the QoL variables to screen for observable multicollinearity. All correlation coefficients were smaller than the pre-decided cut-off level of r=0.75. As a result, all QoL variables were considered in the multivariate analysis. On multivariate analysis, no QoL function or symptom variable was found to be statistically significantly associated with survival. A separate multivariate model was run for global QoL after adjusting for prior treatment history. Global QoL was not found to be significantly associated with survival. VIF values for baseline QoL variables ranged from 1.1 (diarrhea) to 4.3 (fatigue), none of which indicates a significant problem with multicollinearity [22,23]. There was no evidence of non-proportional hazards in the multivariate models presented.

**Table 4:** Baseline QoL measures and associated HRs for death

**Baseline variable**	**QoL score mean (SD)**	**Univariate**		**Multivariate**	
		**HR (95%CI)**	**P**	**HR (95% CI)**	**P**
**General quality of life**					
Global	59.7 (24.2)	1.05 (0.93 – 1.17)	0.45	1.07 (0.94 – 1.19)	0.29
**General function**					
Physical	76.6 (22.8)	0.93 (0.79 – 1.06)	0.27	1.07 (0.80 – 1.35)	0.63
Role	66.9 (31.5)	0.99 (0.90 – 1.07)	0.76	0.91(0.75 – 1.08)	0.30
Emotional	70.1 (23.8)	1.02(0.90 – 1.14)	0.76	1.07 (0.88 – 1.27)	0.48
Cognitive	78.3 (22.8)	0.97 (0.85 – 1.09)	0.66	0.91 (0.71 – 1.11)	0.38
Social	66.5 (32.9)	1.02 (0.93 – 1.11)	0.71	1.02 (0.88 – 1.17)	0.76
**General symptom**					
Fatigue	40.0 (28.4)	1.02 (0.92 – 1.12)	0.76	1.11 (0.87 – 1.35)	0.38
Nausea/vomiting	13.1 (22.4)	1.10 (0.94 – 1.25)	0.21	1.16 (0.97 – 1.35)	0.10
Pain	28.4 (28.5)	1.02 (0.91 – 1.13)	0.70	0.99 (0.83 – 1.15)	0.90
Dyspnea	21.4 (27.9)	1.09 (0.99 – 1.18)	0.07	1.10 (0.92 – 1.27)	0.28
Insomnia	38.4 (34.0)	0.98 (0.89 – 1.07)	0.67	0.90 (0.78 – 1.02)	0.09
Appetite loss	20.6 (29.1)	0.98 (0.89 – 1.07)	0.65	0.90 (0.75 – 1.05)	0.19
Constipation	22.1 (28.9)	0.97 (0.87 – 1.07)	0.55	0.94 (0.80 – 1.08)	0.38
Diarrhea	15.0 (24.5)	0.96 (0.83 – 1.09)	0.58	0.88 (0.72 – 1.04)	0.14
Financial	33.5 (32.7)	0.98 (0.89 – 1.06)	0.58	0.98 (0.86 – 1.09)	0.68

• HRs correspond to a 10-point increment for QoL scores.

• 2 sets of multivariate models were constructed: one for global QoL and other for all general function and symptom variables combined.

• Multivariate model (for general function and symptom variables combined) adjusted for prior treatment history and all baseline QoL variables.

• Multivariate model for global QoL adjusted for prior treatment history.

In order to further investigate the stability of the classical multivariate Cox models reported in Table 4, we conducted a bootstrap resampling procedure based on 1000 samples. The bootstrap estimates of the multivariate HRs along with corresponding p values and 95% BCa CIs are provided in Table 5. We found no significant differences in regression coefficients and their corresponding p values between the classical Cox regression and bootstrap Cox regression models.

**Table 5:** Bootstrap multivariate HRs for baseline QoL measures

**Baseline variable**	**HR (BCa 95% CI)**	**P**
**General quality of life**		
Global	1.07 (0.93 – 1.24)	0.32
**General function**		
Physical	1.07 (0.67 – 1.58)	0.65
Role	0.91(0.64 – 1.15)	0.42
Emotional	1.07 (0.71 – 1.53)	0.53
Cognitive	0.91 (0.65 – 1.13)	0.43
Social	1.02 (0.80 – 1.30)	0.80
**General symptom**		
Fatigue	1.01 (0.75 – 1.73)	0.50
Nausea/vomiting	1.16 (0.64 – 1.59)	0.29
Pain	0.99 (0.69 – 1.23)	0.92
Dyspnea	1.10 (0.83 – 1.42)	0.39
Insomnia	0.90 (0.74 – 1.01)	0.15
Appetite loss	0.90 (0.64 – 1.08)	0.28
Constipation	0.94 (0.72 – 1.12)	0.43
Diarrhea	0.88 (0.58 – 1.11)	0.31
Financial	0.98 (0.75 – 1.20)	0.75

• HRs correspond to a 10-point increment for QoL scores.

• 2 sets of multivariate models were constructed: one for global QoL and other for all general function and symptom variables combined.

• Multivariate model (for general function and symptom variables combined) adjusted for prior treatment history and all baseline QoL variables.

• Multivariate model for global QoL adjusted for prior treatment history.

### Association between changes in quality of life and survival

Table 6 describes the change in scores from baseline to 3 months for all dimensions of EORTC QLQ-C30 instrument. On average, they were small. Table 6 also displays the results of univariate and multivariate Cox regression analyses for change in QoL scores. On univariate analysis, the only QoL scale that was significantly predictive of survival was global function. Before proceeding with multivariate analysis, we checked the bivariate Pearson’s correlation among the change scores to screen for observable multicollinearity. All correlation coefficients were smaller than the pre-decided cut-off level of r=0.75. As a result, all QoL change variables were considered in the multivariate analysis. On multivariate analysis, change variables that were significantly predictive of survival were appetite loss and constipation. The corresponding HRs for 10-point increase (deterioration) in appetite loss and constipation scales were 1.20 (95% CI: 1.06 to 1.35; p=0.005) and 1.13 (95% CI: 1.02 to 1.24; p=0.02) respectively. A separate multivariate model was run for change in global QoL after adjusting for prior treatment history. Every 10-point increase (improvement) in global function from baseline to 3 months was associated with a 10% decreased risk of death (HR=0.90; 95% CI: 0.81 to 0.99, p=0.03). VIF values for change in QoL variables ranged from 1.2 (change in diarrhea) to 3.9 (change in fatigue), none of which indicates a significant problem with multicollinearity. There was no evidence of non-proportional hazards in the multivariate models presented.

**Table 6:** Change in QoL measures and associated HRs for death

**Change variable**	**QoL change mean (SD)**	**Univariate**		**Multivariate**	
		**HR (95% CI)**	**P**	**HR (95% CI)**	**P**
**General quality of life**					
Global	−0.5 (31.1)	0.89 (0.81 – 0.98)	0.01*	0.90 (0.81 – 0.99)	0.03*
**General function**					
Physical	−1.2 (25.6)	1.0 (0.90 – 1.10)	0.99	1.03 (0.82 – 1.23)	0.79
Role	0.0 (36.6)	0.98 (0.92 – 1.05)	0.67	1.11 (0.95 – 1.28)	0.18
Emotional	1.0 (32.2)	0.97 (0.88 – 1.06)	0.48	0.94 (0.78 – 1.09)	0.43
Cognitive	−1.2 (31.2)	1.02 (0.93 – 1.10)	0.69	1.18 (0.89 – 1.33)	0.23
Social	−0.9 (39.9)	0.97(0.91 – 1.03)	0.36	0.90 (0.79 – 1.01)	0.09
**General symptom**					
Fatigue	2.1 (31.0)	1.02 (0.94 – 1.11)	0.58	0.96 (0.73 – 1.19)	0.72
Nausea/vomiting	1.2 (33.1)	1.02 (0.92 – 1.12)	0.69	0.80 (0.75 – 1.03)	0.08
Pain	1.3 (35.4)	1.02 (0.93 – 1.10)	0.71	0.97 (0.83 – 1.11)	0.68
Dyspnea	−0.2 (36.0)	0.97 (0.90 – 1.05)	0.50	0.97 (0.85 – 1.10)	0.66
Insomnia	−4.6 (38.8)	1.02 (0.95 – 1.09)	0.62	1.08 (0.97 – 1.19)	0.15
Appetite loss	0.9 (41.8)	1.06 (1.0 – 1.12)	0.06	1.20 (1.06 – 1.35)	0.005*
Constipation	1.2 (34.8)	1.06 (0.98 – 1.14)	0.12	1.13 (1.02 – 1.24)	0.02*
Diarrhea	−4.3 (28.5)	1.02 (0.91 – 1.13)	0.74	1.07 (0.93 – 1.20)	0.33
Financial	2.4 (39.7)	0.96 (0.90 – 1.03)	0.29	0.88 (0.78 – 1.02)	0.08

• HRs correspond to a 10-point increment for QoL scores.

• 2 sets of multivariate models were constructed: one for global QoL and other for all general function and symptom variables combined.

• Multivariate model (for change in general function and symptom variables combined) adjusted for prior treatment history and all QoL change variables.

• Multivariate model for change in global QoL adjusted for prior treatment history.

• *P <0.05.

In order to further investigate the stability of the classical multivariate Cox models reported in Table 6, we conducted a bootstrap resampling procedure based on 1000 samples. We found no significant differences in regression coefficients and their corresponding p values between the classical Cox regression and bootstrap Cox regression models except for the constipation symptom which changed from being statistically significant in the classical model to being marginally significant in the bootstrap model (results not shown in the interest of space).

We decided to explore the data further by conducting separate multivariate analysis for previously treated patients. Similar to what we observed in the total sample above, improvement in appetite, constipation and global health scores during the first 3 months of treatment was significantly associated with improved survival time in previously treated patients.

## Discussion

Women diagnosed with ovarian cancer often receive therapy over extended periods of time with multiple treatment regimens. Both the acute and chronic effects of the disease and its treatment are associated with significant side-effects that can adversely impact QoL [25]. And while it is expected that QoL of patients will influence survival, identifying the most important factors in ovarian cancer given its heterogeneity with respect to biology, natural history, and multidisciplinary management is a daunting challenge. In fact, it might be expected that it would be easier to uncover QoL elements associated with survival by investigating parameters that are affected, positively or negatively, as a result of therapy. Therefore, the goal of this study was to evaluate the association between changes in QoL during treatment and overall survival in patients with either newly diagnosed or newly-relapsed ovarian cancer.

We found that significant changes in appetite and constipation symptoms, and in global QoL status within 3 months of beginning treatment were predictive of survival time. This is distinct from previous studies wherein association between baseline QoL and survival was explored in ovarian cancer [12-14]. In the study by Carey et al. in advanced ovarian cancer, baseline global QoL EORTC QLQ-C30 score was found to be an independent predictor for both progression-free and overall survival. Baseline EORTC QLQ-C30 cognitive functioning score was also an additional independent predictor for overall survival. In addition, at 3 months after completion of chemotherapy, global QoL score, performance status and grade were significant independent predictors of overall survival [12]. In a previous study conducted by our research team, baseline QoL was evaluated using Ferrans and Powers Quality of Life Index in 90 ovarian cancer patients. The health and physical domain was found to be significantly (although marginally) associated with survival [13]. Finally, in a recently published study by von Gruenigen et al., conducted in 399 stage III ovarian cancer patients receiving adjuvant chemotherapy, QoL was assessed using the Functional Assessment of Cancer Therapy-General. Poor physical well-being reported at baseline was found to be associated with a significantly increased risk of death [14]. The present study builds on this previous research to explore whether the dynamic effects of therapy on QoL scores (as opposed to a single baseline assessment) is associated with survival in ovarian cancer patients.

The results of this study suggest that baseline QoL should be considered when planning treatment and regular QoL assessment performed during the course of treatment in women with ovarian cancer. Moreover, particular attention should be paid to QoL parameters related to global health, appetite and constipation and, when indicated, suitable interventions to support these parameters should be applied. Positive effects on survival as a consequence of such interventions would go a long way towards establishing causative relationships between these specific QoL parameters and disease control.

Unfortunately, while there has been some progress with respect to the treatment of appetite loss in cancer patients, clinical effectiveness is inconsistent and unpredictable. And there are at present no effective means to address more complex QoL factors such as global health. This challenges the cancer research enterprise to develop greater understanding of the complex physiology responsible for all aspects of QoL, and to use this information to develop more effective and predictable methods to favorably modulate this critical aspect of patient health and wellness.

Several limitations of this study need to be acknowledged. Our study, because of its retrospective nature, relies on data not collected to test a specific hypothesis. As a result, we could not control for certain factors in our analyses that could influence survival such as treatment received, medical co-morbidities, socioeconomic factors, support system, exercise and educational level. The patient cohort was limited only to those patients who were English speakers and therefore is not representative of the complete spectrum of ovarian cancer patients. A majority of our patients had advanced stage disease at presentation and had failed primary treatment elsewhere before coming to our hospital. As a result, we acknowledge that our findings may not be applicable to newly-diagnosed ovarian cancer patients with limited stage disease, an issue that needs to be tested in suitable patient populations.

Moreover, this study does not reveal a causative relationship between QoL and survival. Rather, patient QoL was found to act as a surrogate for otherwise undetected prognostic factors [26]. QoL scores were assessed over a 3-month interval only which may not be sufficient time for score changes to develop in other QoL parameters that may be prognostic of survival. We did not control for the multiple comparisons made in this study, but this is acceptable for hypothesis-generating studies [27].

This study also has several strengths, including no missing data on any EORTC QLQ-C30 variables for the entire study sample; the use of a valid and reliable QoL instrument; the availability of clinical parameters in nearly all patients; and availability of mature and reliable survival data. As is the case for all exploratory retrospective studies, the most important outcome that can be achieved is the development of a hypothesis suggested by the results. As a consequence of this study, we hypothesize that the parameters of appetite loss, constipation and global health are independent determinants of survival in ovarian cancer, and should be regularly assessed and when indicated, targeted for intervention.

## Conclusions

This exploratory study provides preliminary evidence to indicate that ovarian cancer patients whose appetite, constipation and global health improve within 3 months of treatment have a significantly increased probability of survival. Given that QoL is as meaningful as the actual length of life in patients with advanced ovarian cancer, these findings should be used in clinical practice to systematically address QoL-related problems of ovarian cancer patients throughout their treatment course.

## Competing interests

The authors declare that they have no competing interests.

## Authors’ contributions

DG and DPB participated in concept, design, data collection, data analysis, data interpretation and writing. EDS participated in concept, design, data interpretation and writing. MM participated in data interpretation and writing. All authors read and approved the final manuscript.
